# Long non-coding RNA TCONS_00000200 as a non-invasive biomarker in patients with intracranial aneurysm

**DOI:** 10.1042/BSR20182224

**Published:** 2019-11-22

**Authors:** Chenghan Wu, Hailong Song, Yinzhou Wang, Lili Gao, Yali Cai, Qiong Cheng, Yanru Chen, Zheng Zheng, Yuansheng Liao, Jushan Lin, Buni Xie, Weiwu Cai, Shiju Li, Lianming Liao, Xiaohua Yan

**Affiliations:** 1 Department of Neurology, Second Affiliated Clinical College of Fujian University of Traditional Chinese Medicine, Fuzhou 350001, China; 2 Department of Neurosurgery, Penn Center for Brain Injury and Repair, Perelman School of Medicine, University of Pennsylvania, Philadelphia, PA 19104, U.S.A.; 3 Department of Neurology and Traditional Chinese Medicine, Fujian Provincial Hospital, Fuzhou 350001, China; 4 Department of Neurology, Quanzhou hospital of Traditional Chinese Medicine, Quanzhou 362000, China; 5 Central Laboratory, The Union Hospital of Fujian Medical University, Fuzhou 350001, China

**Keywords:** biomarker, intracranial aneurysm, lncRNA, microarray, mRNA

## Abstract

We performed long non-coding RNA (lncRNA) microarray assay to identify lncRNAs with differential expression between patients with intracranial aneurysm (IA) and healthy control individuals to evaluate their potential use as biomarkers of IA. Arraystar Human lncRNA Microarray v3.0 was performed to identify differentially expressed lncRNAs and mRNAs in plasma samples (4 ml). lncRNAs with the most pronounced differential expression were used to select gene markers, and results were validated by quantitative reverse-transcription polymerase chain reaction (qRT-PCR). Plasma levels of *TCONS_00000200* (fold change: 2.28) and *ENST00000511927* (fold change: 2.50) were significantly higher in IA patients than in healthy individuals (*P*<0.001), and plasma levels of *ENST00000421997* (fold change: 0.45) and *ENST00000538202* (fold change: 0.43) were significantly lower in IA patients than in healthy individuals (*P*<0.001). qRT-PCR confirmed the same trends of up- and down-regulation of these four lncRNAs. A receiver operating characteristic (ROC) curve for *TCONS_00000200* showed that the area under the curve (AUC) was 0.963 (95% confidence interval, 0.919–1.000), optimal cut-off point was 0.0081, sensitivity was 90.0%, and specificity was 96.7%. These results indicate that the lncRNA *TCONS_00000200* is differentially expressed in the plasma of IA patients and could serve as a biomarker of IA.

## Introduction

Intracranial aneurysm (IA) is a tumour-like structural abnormality formed after pathological dilation and expansion of the cerebral vessel wall. IA rupture can directly result in subarachnoid or cerebral haemorrhage, which has high disability and mortality rates. Therefore, IA is a life-threatening condition, and early detection is critical for successful treatment. However, IA may be asymptomatic before rupture and haemorrhage. In rare cases, IA is detected on brain computed tomography angiography (CTA) or magnetic resonance angiography (MRA) during routine check-up, allowing intervention or surgery to effectively prevent cerebral haemorrhage.

IA is associated with congenital dysplasia and heredity factors. Therefore, genetic screening of IA-related genes using a small venous blood sample could indicate the need for further brain CTA, MRA, or digital subtraction angiography. The search for causative genes is now possible due to advances in high-throughput genomic technology. In particular, long non-coding RNAs (lncRNAs), which are >200 nucleotides in length, play important roles in regulating gene expression at transcriptional and post-transcriptional levels. As novel biomarkers with potential clinical applications, lncRNAs have the following advantages: (1) they are expressed in peripheral blood [1]; (2) their abnormal expression is an indicator of several medical conditions and disease states; (3) their expression has tissue, spatiotemporal, and disease specificity indicative of certain disease stages or types [2]; and (4) they are being explored as targets of drug therapy for neurological tumours [3]. Thus, lncRNAs are attracting much attention from researchers and hold great promise for clinical applications.

## Methods

The present study was approved by the Ethics Committee of the Second Affiliated Clinical College of Fujian University of Traditional Chinese Medicine and was carried out in accordance with the Declaration of Helsinki and other relevant guidelines and regulations.

### Subjects

Patients admitted to the Second People’s Hospital of Fujian University of Traditional Chinese Medicine or Fujian Provincial Hospital between February 2014 and June 2016 for subarachnoid haemorrhage underwent brain digital subtraction angiography. Five patients were found to have IA after a first screening (Table 1), and an additional 30 patients were found to have IA after a second screening (Table 2). All patients met the diagnostic criteria of IA [4] and provided informed consent.

**Table 1:** Clinical characteristics of the individuals included in the study (first screening)

Items	IA (*n*=5)	Control (*n*=4)
Gender (female, %)	3 (60.00%)	2 (50.00%)
Age (years, mean ± SD)	52.40 ± 8.59	51.72 ± 7.28
Hypertension, %	4 (80.00%)	75.00%)
Smokers, %	2 (40.00%)	2 (50.00%)
Drinkers, %	2 (40.00%)	1 (25.00%)
Drugs, %	0	0
History of other aneurysms, %	0	0

No significant between-group differences were observed in the general information (*P*>0.05).

**Table 2:** Clinical characteristics of the individuals included in the study (second screening)

Items	IA (*n*=30)	Control (*n*=20)
Gender (female,%)	19 (63.33%)	13 (65.00%)
Age (years, mean ± SD)	53.60 ± 12.72	53.60 ± 12.71
Hypertension, %	17 (56.66%)	11 (55.00%)
Smokers, %	6 (20.00%)	4 (20.00%)
Drinkers, %	5 (16.66%)	3 (15.00%)
Drugs, %	0	0
History of other aneurysms, %	0	0

No significant between-group differences were observed in the general information (*P*>0.05).

Healthy individuals who visited the Second People’s Hospital of Fujian University of Traditional Chinese Medicine between February 2014 and June 2016 were recruited as a control group. Individuals with no signs of stroke or cerebrovascular abnormalities per brain CT or CTA and who had comparable age and sex as the IA patients were enrolled. Four healthy individuals were selected after a first screening (Table 1), and an additional 20 healthy individuals were selected after a second screening (Table 2).

### Arraystar lncRNA array protocol

**Figure 1:**
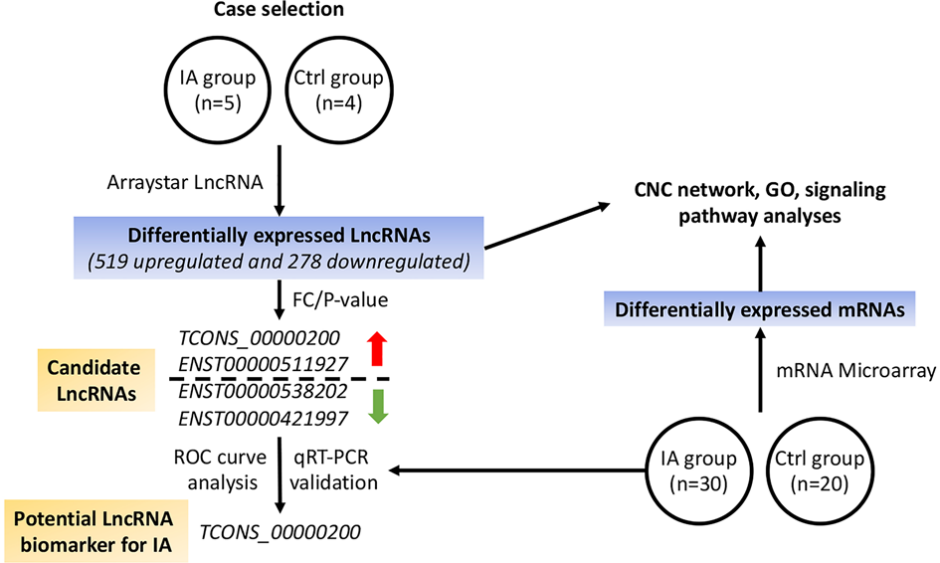
Schematic workflow of the study We performed Arraystar LncRNA profiling and analysis and further validated candidate lncRNAs using samples from additional patients. CNC network, GO, and KEGG pathway analyses were also performed.

The study was conducted according to the procedures shown in Figure 1.

### Sample collection

A peripheral venous blood sample (4 ml) was collected from each subject and placed in a 5-ml EDTA-2K vacuum tube, which was centrifuged at 1000×***g*** for 10 min at room temperature to separate the plasma and cellular components. After separation, 500 µl plasma was added to the RNA preservation solution (Bioteke) and stored at -80°C for later testing.

### Total RNA extraction and RNA quality testing

Total RNA was extracted using TRIzol (Life Technologies; cat. no. 10296-028) according to the manufacturer’s protocol and as previously described [5]. Briefly, a 250-μl plasma sample was acquired by centrifuging at 12000×***g*** for 10 min at 4°C. Next, 750 μl TRIzol LS reagent was added to the sample, and the 1.5-ml tube was shaken and incubated for 5 min at 15–30°C to ensure that nucleic acid protein complexes were completely dissociated. After the addition of 0.2 ml chloroform, the sample was shaken for 15 s and incubated for 2–3 min at room temperature. The mixture was further centrifuged at 12000×***g*** for 15 min at 4°C. The aqueous phase of the sample was extracted (∼60%) and transferred to a new tube. Next, 500 μl of 100% isopropanol was added to the aqueous phase, which was incubated for 10 min at 15–30°C and centrifuged at 12000×***g*** for 10 min at 4°C. The supernatant was removed, and the RNA pellet was washed with 1 ml of 75% ethanol, briefly vortexed, and centrifuged at 7500×***g*** for 5 min at 4°C. The RNA pellet was then dried for 5–10 min at room temperature. Finally, the RNA pellet was resuspended in RNase-free water and incubated in a water bath at 55–60°C for 10 min. The sample was stored in a −70°C refrigerator. Total RNA purity and integrity were determined using a NanoDrop ND-1000 spectrophotometer (NanoDrop Products, Wilmington, DE).

### RNA labeling and microarray hybridisation

Complementary DNA was labelled with an Arraystar RNA Flash Labeling Kit, purified with an RNeasy Mini Kit (Qiagen), and hybridised with an Arraystar Human LncRNA Microarray V3.0 (8×6 k, Arraystar Inc., Rockville, MD, U.S.A.) under standard conditions. The lncRNA microarray could detect 30586 lncRNAs and 26109 protein-encoding transcripts. lncRNAs were selected from recognised public transcriptome databases, including Refseq, UCSC Known Gene, and Gencode.

### Data collection and normalisation

An Agilent scanner was used to perform the microarray scan. The quantile algorithm of Gene Spring v13.0 (Agilent Technologies, CA, U.S.A.) was used to normalise the raw data. Differentially expressed lncRNAs were selected and analysed. Microarray data are available in the Gene Expression Omnibus (GEO) database (GSE75436; http://www.ncbi.nlm.nih.gov/geo). R software v3.2.3 was used for array analysis. Differentially expressed lncRNAs were selected for analysis of gene markers with abnormal (i.e. differential) expression.

### Coding-non-coding analysis

Coding gene data were normalised, and data for different transcripts of the same gene were used to calculate the median value as representative of gene expression. Normalised data for the selected lncRNAs and all genes were used to calculate correlation coefficients; records with a |Pearson correlation coefficient| value ≥ 0.9 and *P*-value ≤ 0.05 were selected. Selected records were used to graph the Coding-non-coding (CNC) network using Cytoscape v2.8.3.

### Quantitative reverse-transcription polymerase chain reaction

Quantitative reverse-transcription polymerase chain reaction (qRT-PCR) was performed to verify gene markers with abnormal (i.e., differential) expression according to instructions provided with the TRIzol LS reagent (Invitrogen Life Technologies, U.S.A.) using the 30 IA patients and 20 healthy individuals from the second screening. Primer sequences of lncRNAs and β-actin are shown in Table 3.

### Statistical analysis

SPSS v20.0 (SPSS Inc., Chicago, IL, U.S.A.) was used for statistical analysis. Independent samples *t* tests and Fisher’s exact tests were performed, with significant *P*-values defined as <0.05. Agilent Feature Extraction v11.0.1.1 was used to capture the microarray map, read the values, and obtain the raw data, which were processed with GeneSpring GX v12.1 (Agilent Technologies) for quantile normalisation and data processing to select high-quality probes for analysis. Differentially expressed lncRNAs were filtered based on *t* test *P*-values (|fold change| ≥ 2.0, *P*-value <0.05). Receiver operating characteristic (ROC) curves were used to analyse the predictive performance of differentially expressed lncRNAs and the optimal cut-off points for IA patients. The Youden index (J = sensitivity + specificity − 1) was also used to assess the predictive performance of the lncRNAs. CNC, Gene Ontology (GO), and Kyoto Encyclopaedia of Genes and Genomes (KEGG) pathway analyses were also performed.

## Results

### Differentially expressed lncRNAs in plasma samples

**Figure 2:**
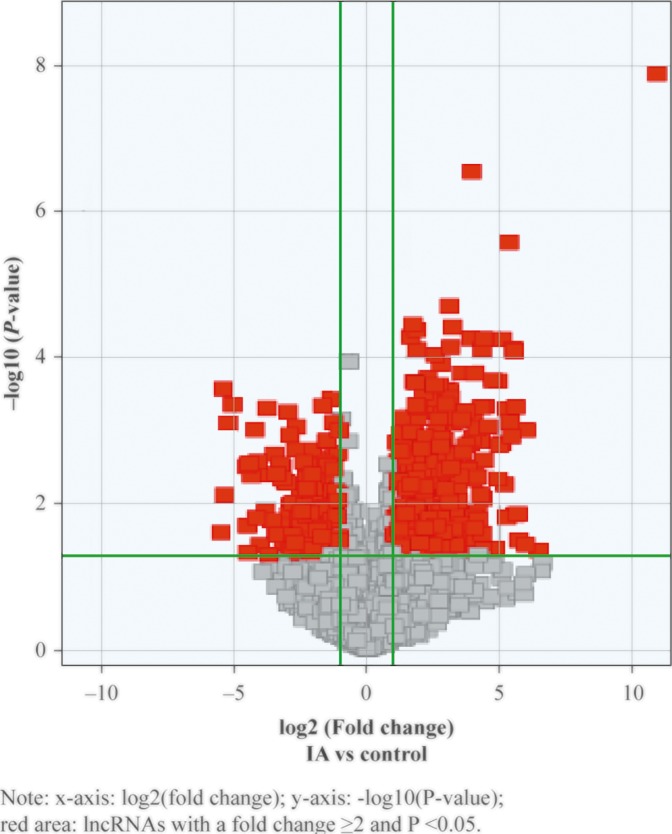
Volcano maps for IA patients and healthy control individuals x-axis: log2 (fold change); y-axis: −log10 (*P*-value); red area: lncRNAs with |fold change| ≥ 2 and *P*<0.05.

Microarray assay was used to measure the plasma levels of lncRNAs in IA patients and healthy individuals and select differentially expressed lncRNAs (criteria: *P*<0.05, |fold change| ≥ 2.0) (Figure 2), which included 519 up-regulated and 278 down-regulated lncRNAs. Differentially expressed lncRNAs were further analysed to investigate the ten lncRNAs with the largest differential expression (considering multiple factors including fold change and *P*-value) (Table 3).

**Table 3:** lncRNAs with the most pronounced differential expression in IA patients

*P*-value	FDR	Fold change	Regulation	Type	Seqname	Gene symbol	Source
0.000477976	0.075081743	48.5505021	up	Noncoding	TCONS_00013319	XLOC_005968	LincRNAs identified by Cabili et al.
7.68221E-05	0.0369362	45.8398828	up	Noncoding	ENST00000511927	RP11-504G3.1	GENCODE
2.64818E-06	0.006340623	40.2860164	up	Noncoding	TCONS_00000200	XLOC_000199	LincRNAs identified by Cabili et al.
5.70695E-05	0.0369362	33.8583896	up	Noncoding	TCONS_00010726	XLOC_004448	LincRNAs identified by Cabili et al.
0.00021227	0.060381322	30.4468496	up	Noncoding	TCONS_00018266	XLOC_008547	LincRNAs identified by Cabili et al.
0.000270567	0.064243696	43.4186222	down	Noncoding	ENST00000538202	RP11-429A20.4	GENCODE
0.000441893	0.075081743	33.8474532	down	Noncoding	ENST00000421997	RP11-86H7.1	GENCODE
0.000498757	0.075081743	13.7918859	down	Noncoding	ENST00000563397	RP11-525K10.1	GENCODE
0.002841449	0.156844354	21.9588842	down	Noncoding	TCONS_00004595	XLOC_002498	LincRNAs identified by Cabili et al.
0.002144808	0.13167657	11.3773563	down	Noncoding	ENST00000424968	LINC00240	GENCODE

**Table 4:** Primer sequences of lncRNAs and β-actin

Gene	Primer Sequences (5′→3′)
*β-actin*	F: 5′ GTGGCCGAGGACTTTGATTG 3′
	R: 5′ CCTGTAACAACGCATCTCATATT 3′
*ENST00000511927*	F: 5′ GGTGCAAGCAGTCTTCCCATCT 3′
	R: 5′ TGTGCCTGAGGATCAGCTACCC 3′
*ENST00000421997*	F: 5′ TGACGCTGAGTGAAAGAATGC 3′
	R: 5′ GAGCACGGATGACCTTGAAG 3′
*TCONS_00000200*	F: 5′ GGTCACATTCACCCTCCTGC 3′
	R: 5′ TCACCTCGTTTGCTTCCTGTT 3′
*ENST00000538202*	F: 5′ AGGTAGAATTACCATATCCCACTC 3′
	R: 5′ GCTGCCTGCTACATATTTGACT 3′

### qRT-PCR validation of lncRNAs

**Figure 3:**
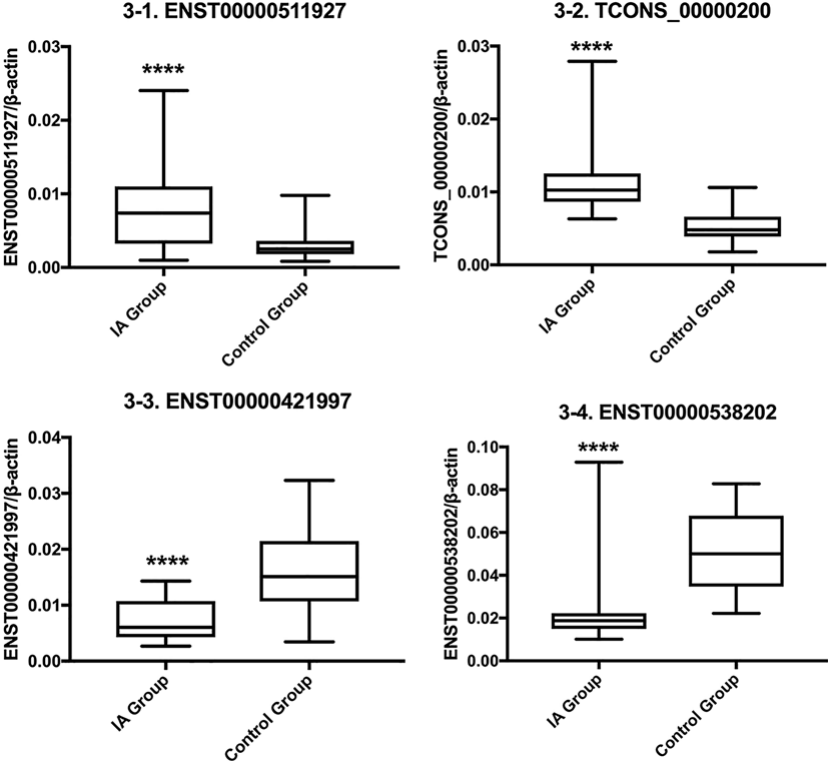
Differences in plasma lncRNA expression between IA patients and healthy control individuals y-axis represents the relative expression of corresponding lncRNAs (min-max). ****Significant differences.

We initially selected the top ten lncRNAs for validation from a small number of subjects in the first screening. As no significant differences in the expression of six lncRNAs (including *TCONS_00013319*) were detected among the larger number of subjects in second screening, these lncRNAs were not further validated. The four lncRNAs that underwent further validation showed consistent up- or down-regulation among subjects in the second screening. Therefore, qRT-PCR was performed to validate the four lncRNAs with the most pronounced differential expression: *TCONS_00000200* (up), *ENST00000511927* (up), *ENST00000538202* (down), and *ENST00000421997* (down) (Figure 3). Plasma levels of *TCONS_00000200* and *ENST00000511927* were significantly higher in IA patients than in healthy individuals (*P*<0.01), whereas plasma levels of *ENST00000421997* and *ENST00000538202* were significantly lower in IA patients than in healthy individuals (*P*<0.01). Thus, qRT-PCR showed consistent up- and down-regulation trends for these four lncRNAs as in the microarray assay. One additional lncRNA, *ENST00000494340*, which showed evidence of up-regulation in IA patients in the first screening was further tested among a larger number of subjects in the second screening, but no significant between-group differences were observed (*P*>0.05; Table 5 and Figure 3).

**Table 5:** qRT-PCR results for lncRNAs

LncRNA	qRT-PCR				
	IA group a	Control group b	IA group/control group	*P*-value	Up/down-regulation
TCONS_00000200	1.19 × 10^-2^	5.21 × 10^-3^	2.28	<0.001	Up
ENST00000511927	8.06 × 10^-3^	3.23 × 10^-3^	2.50	<0.001	Up
ENST00000421997	7.19 × 10^-3^	1.59 × 10^-2^	0.45	<0.001	Down
ENST00000538202	2.17 × 10^-2^	4.99 × 10^-2^	0.43	<0.001	Down
ENST00000494340	7.35 × 10^-3^	8.54 × 10^-3^	0.86	0.390	Up

‘IA group/control group’ indicates the relative fold change of an lncRNA in IA patients compared with healthy individuals.

a represents relative mean expression per qRT-PCR in the IA group with hemorrhage and stroke; b represents relative mean expression per qRT-PCR in the control group. IA group/control group means the relative fold change of an lncRNA in the IA group as compared with the control group.

### ROC curves

**Figure 4:**
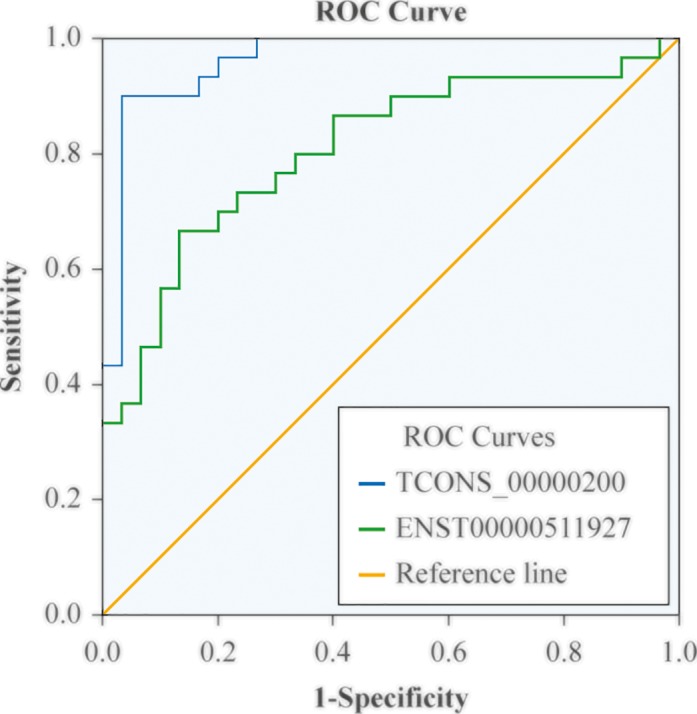
ROC curves of TCONS_00000200 and ENST 00000511927

ROC curves were constructed based on all subjects. *TCONS_00000200* and *ENST00000511927*, the two lncRNAs with the most pronounced differential expression (i.e., up-regulated in IA patients; *P*<0.01) were analysed to investigate their potential use as biomarkers of IA. The ROC curve of the plasma level of *TCONS_00000200* showed that the area under the curve (AUC) was 0.963 (95% confidence interval (CI), 0.919–1.000), optimal cut-off point was 0.0081, sensitivity was 90.0%, and specificity was 96.7% (J = 0.867). The ROC curve of the plasma level of *ENST00000511927* showed that the AUC was 0.804 (95% CI, 0.696–0.920), optimal cut-off point was 0.04615, sensitivity was 66.7%, and specificity was 86.7% (J = 0.534). The sensitivity and specificity of *TCONS_00000200* were higher than those of *ENST00000511927* (Figure 4), suggesting that the plasma level of *TCONS_00000200* might better serve as a biomarker of IA.

### CNC analysis

**Figure 5:**
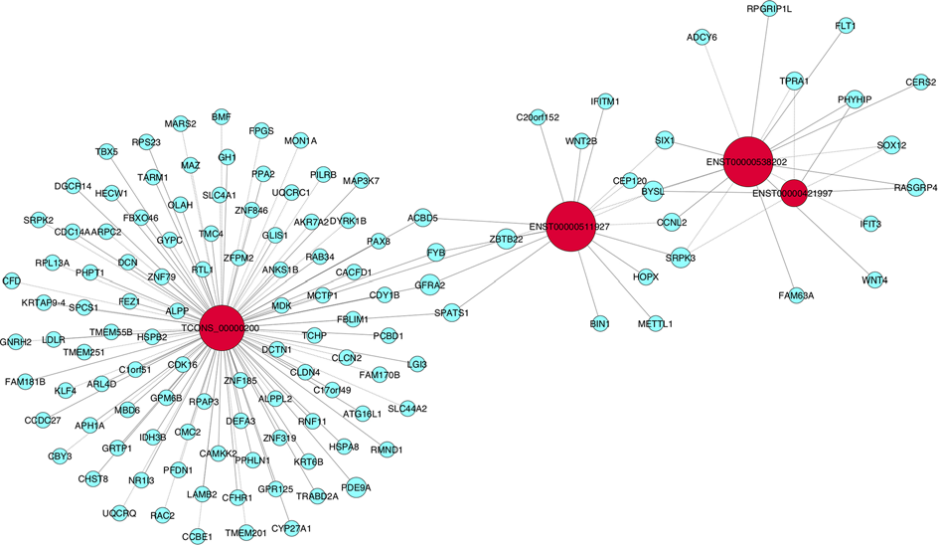
CNC network Red nodes represent lncRNAs, blue nodes represent coding genes, solid lines represent positive correlations, and dotted lines represent negative correlations. Node size is proportional to node degree.

The four lncRNAs with significant differential expression (*ENST00000538202, ENST00000511927, ENST00000421997*, and *TCONS_00000200*) and differentially expressed mRNAs identified by microarray assay were included in CNC analysis. All mRNAs with differential expression between IA patients and healthy individuals were selected (Figure 5). Correlation coefficients between the relevant gene and lncRNA are shown in Table 6 and Supplementary Table S1.

**Table 6:** CNC analysis correlation coefficients between the relevant gene and lncRNA

Gene	LncRNA	Correlation coefficient	Correlation
*CD276*	ENST00000511927	0.79447961	+
*SPN*	ENST00000511927	0.813826832	+
*HAVCR2*	ENST00000511927	0.794300016	+
*HECW1*	TCONS_00000200	0.790902277	+
*OLAH*	TCONS_00000200	0.929005697	+

### GO and KEGG pathway analyses

**Figure 6:**
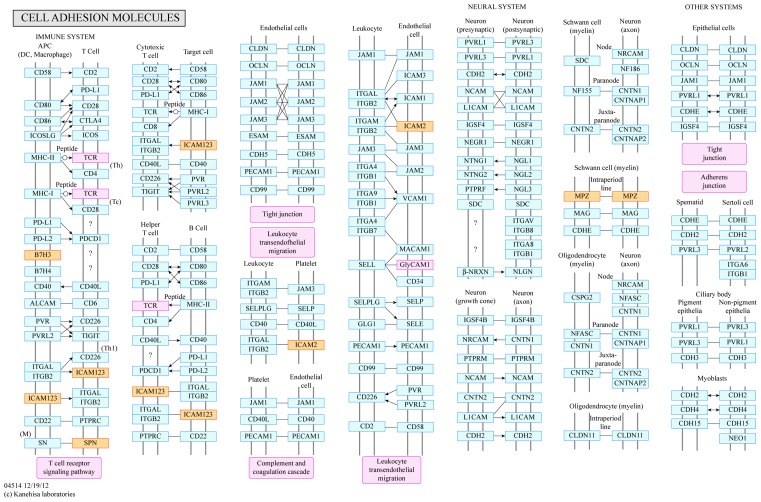
KEGG pathway analysis of related metabolic pathways Note that CD276 is also known as B7-H3. Up-regulated gene markers (orange) are involved in pathways such as T-cell receptor signalling and leucocyte transendothelial migration.

Differentially expressed mRNAs from CNC analysis were used for GO and KEGG pathway analyses. GO analysis showed that the differentially expressed mRNAs were mainly involved in the following molecular functions: ATPase-modulating activity, organophosphate hydrolase activity, protein tyrosine/serine/threonine phosphatase activity, and fibroblast growth factor receptor binding. Cellular components mainly included the Golgi, cytoplasm, sarcomeres, transverse tubules, myofibrils, and myosin. Biological processes mainly included negative regulation of muscle tissue development, negative regulation of striated muscle tissue development, skeletal muscle development, organ development, skeletal muscle contraction, ATP hydrolysis-coupled membrane transport, lymphocyte-mediated negative immune regulation, and T-cell activation and negative regulation. The top five GO entries based on enrichment score are listed in Supplementary Table S2, and the top ten GO entries based on enrichment score related to biological processes are listed in Supplementary Table S3. Fifteen GO entries were related to immune inflammatory responses in biological processes (Supplementary Table S4). KEGG pathway analysis identified five metabolic pathways associated with the differentially expressed genes (Supplementary Table S5 and Figure 6).

## Discussion

Okazaki et al. [6] first identified lncRNA while performing large-scale sequencing of the mouse full-length complementary DNA library. With advances in microarray assay and sequencing technology [7], lncRNAs are now attracting a great deal of attention from researchers. In particular, the ‘central dogma’ of molecular biology is now being questioned by the research community. That is, in the past, researchers believed that RNA acted as the intermediary for the transfer of genetic information between DNA and proteins. However, findings from the Human Genome Project revealed that only 1.5% of the 3 billion base pairs in the human genome encode proteins, with the remaining 98.5% being non-protein coding sequences were once considered unwanted ‘junk sequences’ that accumulated during genetic evolution [8]. However, further research showed that these ‘junk sequences’ have important biological functions in normal cells [7]. In 2012, the Encyclopaedia of DNA Elements (ENCODE) study showed that most non-protein coding sequences are lncRNAs located in the nucleus or cytoplasm that exhibit tissue- or spatiotemporal-specific expression [2] but do not or rarely encode protein [9]. These lncRNAs regulate gene expression at the epigenetic, transcription, and post-transcription levels [10,11] and are closely related to human growth and development as well as disease onset and progression. Also, lncRNAs could target mRNA and thereby regulate different degrees of RNA transcription.

lncRNAs play important roles in the development of cardiovascular diseases by regulating endothelial cell and smooth muscle cell (SMC) functions, SMC proliferation, and atherosclerosis [12,13]. lncRNAs expressed in endothelial cells, such as Tie-1-AS and metastasis-associated lung adenocarcinoma transcript 1, regulate vascular development [14,15]. Bell et al. [16] identified an lncRNA, named smooth muscle and endothelial cell-enriched migration/differentiation-associated lncRNA (SENCR), which controls the contractile phenotype of SMCs and is associated with their migration and differentiation. lncRNA-p21 is involved in the regulation of SMC proliferation and atherosclerotic changes [17]. ANRIL has been identified as a genetic susceptibility locus associated with IA and abdominal aortic aneurysm [18–20]. HIF1a-AS1 is overexpressed in the thoracoabdominal aortic aneurysm and plays a key role in the proliferation and apoptosis of vascular SMCs *in vitro* [21]. The inflammatory response affects mesolamellar vascular SMCs, resulting in energy metabolism disorder, degeneration, apoptosis, and subsequent elastic fibre degeneration and destruction, breakage, and disappearance [22]. Although inflammatory cells do not normally infiltrate the arterial wall, many macrophages and lymphocytes expressing interleukin-6 and tumour necrosis factor-α are found in IA tissue [23] and may contribute to IA formation [24–26]. The arterial wall becomes thinner as it becomes brittle and starts to bulge locally due to elevated intravascular pressure, forming an IA [27]. Li et al. [28] showed that the lncRNA H19 is involved in the development of abdominal aortic aneurysm and that its inhibition could be used to treat aortic aneurysms. These results indicate that lncRNAs are involved in the development of cardiovascular diseases involving endothelial cells, SMCs, and inflammatory responses, including IA.

We found that four differentially expressed lncRNAs and mRNAs in IA patients were associated with sarcomeres, transverse tubules, myofibrils, myosin, ATPase-modulating activity, and regulation of muscle tissue development, suggesting that IA involves the regulation of SMC function. Moreover, GO entries suggested the negative regulation of lymphocyte-mediated immunity and T-cell activation in IA, suggesting its relation to inflammatory responses. Overall CNC, GO, and KEGG pathway analyses showed that IA is associated with complex interactions among many molecular mechanisms, including the involvement of immune-related biological processes and expression of *SPN, CD276*, and *HAVCR2* genes. Although we did not further test the function of these genes and the underlying mechanisms of immune responses in IA, a previous study shows that the pathogenesis of IA may be mediated by proinflammatory cytokines [29]. Further research is needed to investigate the relationship between these genes and the aetiology of IA.

Previous studies show that chromosome 9p21 is a susceptibility site for abdominal aortic aneurysm and IA [30]. In particular, Olsson et al. [31] performed Hapmap tagging and genetic analysis of six single nucleotide polymorphism sites in the 44000 base pair region of 9p21 and found that 9p21 is a susceptibility site for IA and is related to IA rupture. We performed genetic analysis of ten family members from the same lineage of familial IA and identified a novel gene, *PKD1*, on chromosome 16 (data not shown). In the present study, lncRNA microarray analysis identified 519 up-regulated and 278 down-regulated lncRNAs with differential expression related to IA (*P*<0.05, |fold change| ≥ 2.0). Five lncRNAs with the most pronounced differential expression were selected for qRT-PCR verification: *ENST00000494340, TCONS_00000200, ENST00000511927, ENST00000538202*, and *ENST00000421997*. We found that plasma *TCONS_00000200* expression was higher in IA patients than in healthy individuals (*P*<0.01) and an ROC curve showed that its AUC was 0.963 (95% CI, 0.919–1.000), sensitivity was 90.0%, and specificity was 96.7%, indicating that this lncRNA could serve as a biomarker of IA. We confirmed these results through qRT-PCR of venous blood samples from ten family members from the same lineage of familial IA (data not shown). Nevertheless, future studies including larger cohorts of patients and healthy control individuals are needed to determine the usefulness of this lncRNA in IA diagnosis and prognosis prediction. As stated in an article recently published in *Science*, ‘understanding the genetic differences that make us human is a long-standing endeavour that requires the comprehensive discovery and comparison of all forms of genetic variation within great ape lineages’ [32].

## Conclusion

The plasma level of the lncRNA *TCONS_00000200* could serve as a biomarker of IA due to its high sensitivity and specificity.

## Supplementary Material

Supplementary Tables S1-S5

## Abbreviations

ANRIL: Antisense noncoding RNA in the INK4 locus
AUC: area under the curve
CI: confidence interval
CNC: coding-non-coding
CTA: computed tomography angiography
GO: Gene Ontology
HIF1a: Hypoxia-inducible factor 1-alpha
IA: intracranial aneurysm
KEGG: Kyoto Encyclopaedia of Genes and Genomes
lncRNA: long non-coding RNA
MRA: magnetic resonance angiography
qRT-PCR: quantitative reverse-transcription polymerase chain reaction
ROC: receiver operating characteristic
SMC: smooth muscle cell
Tie-1-AS: Tyrosine kinase containing immunoglobulin and epidermal growth factor homology domain 1-antisense
