# Supplementary material for: Long non-coding RNA TCONS_00000200 as a non-invasive biomarker in patients with intracranial aneurysm
Source: Biosci Rep. 2019 Nov 22;39(11):BSR20182224. doi: 10.1042/BSR20182224 (PMC6879357; doi:10.1042/BSR20182224)
Supplement: Supplementary Tables S1-S5 [file BSR-2018-2224_supp.pdf]

Supplemental Table 1-5

Supplemental Table 1: CNC network detailed analysis for the correlation coefficient between the relevant gene and lncRNA

mRNA	LncRNA	PCC	pcc_group	P-value	FDR
DGCR14	TCONS_00000200	0.962121457	+	3.36E-05	0.00375252
HECW1	TCONS_00000200	0.95543427	+	5.89E-05	0.00413941
TRABD2A	TCONS_00000200	0.988102579	+	5.98E-07	0.00049907
PDE9A	TCONS_00000200	0.916945506	+	0.00050085	0.01236248
DCTN1	TCONS_00000200	0.963380613	+	2.99E-05	0.00374754
IFITM1	ENST00000511927	0.901706511	+	0.00088946	0.01604338
OLAH	TCONS_00000200	0.993247509	+	8.28E-08	0.00020358
RPS23	TCONS_00000200	0.924683344	+	0.00035844	0.01064919
ZNF319	TCONS_00000200	0.948058289	+	9.99E-05	0.00575086
FEZ1	TCONS_00000200	0.920187655	+	0.00043711	0.01156231
BIN1	ENST00000511927	0.961567912	+	3.53E-05	0.00377427
ANKS1B	TCONS_00000200	0.900443085	+	0.00092893	0.01604338
HSPB2	TCONS_00000200	0.980727783	+	3.21E-06	0.00098736
LGI3	TCONS_00000200	0.908901536	+	0.00068663	0.01419594
GH1	TCONS_00000200	0.923325579	+	0.00038105	0.01064919
TARM1	TCONS_00000200	0.937035471	+	0.00019386	0.00794817
WNT2B	ENST00000511927	0.923078437	+	0.00038528	0.01064919
RNF11	TCONS_00000200	0.963163823	+	3.05E-05	0.00374754
CYP27A1	TCONS_00000200	0.946986064	+	0.00010723	0.00599495
SRPK3	ENST00000511927	0.916380347	+	0.00051259	0.01236248
SRPK3	ENST00000421997	-0.949499711	-	9.07E-05	0.00557728
SRPK3	ENST00000538202	-0.962330577	-	3.29E-05	0.00375252
ACBD5	ENST00000511927	0.94796943	+	0.00010052	0.00575086
ACBD5	TCONS_00000200	0.932494169	+	0.00024625	0.0087268
CMC2	TCONS_00000200	0.901366441	+	0.00089996	0.01604338
GPR125	TCONS_00000200	0.900327543	+	0.0009326	0.01604338
PHPT1	TCONS_00000200	0.960761471	+	3.79E-05	0.0038866
APH1A	TCONS_00000200	0.957922746	+	4.83E-05	0.00398362
C20orf152	ENST00000511927	0.923416484	+	0.00037951	0.01064919
ARL4D	TCONS_00000200	0.902361637	+	0.00086945	0.01584335
MAP3K7	TCONS_00000200	0.925278997	+	0.00034882	0.01064919

SOX12	ENST00000421997	-0.914105507	-	0.0005618	0.01277452
SOX12	ENST00000538202	-0.902382434	-	0.00086882	0.01584335
GFRA2	ENST00000511927	0.911420562	+	0.00062402	0.01326625
GFRA2	TCONS_00000200	0.957454407	+	5.02E-05	0.00398362
SPATS1	ENST00000511927	0.904029903	+	0.00081994	0.01575813
SPATS1	TCONS_00000200	0.956298311	+	5.50E-05	0.00410302
ADCY6	ENST00000538202	-0.918811277	-	0.00046343	0.01200039
TPRA1	ENST00000421997	-0.95893498	-	4.44E-05	0.00398362
TPRA1	ENST00000538202	-0.95672685	-	5.32E-05	0.00408952
C17orf49	TCONS_00000200	0.923803753	+	0.00037298	0.01064919
PPHLN1	TCONS_00000200	0.924276558	+	0.00036511	0.01064919
CAMKK2	TCONS_00000200	0.928405175	+	0.0003013	0.00988264
PCBD1	TCONS_00000200	0.907621346	+	0.00072007	0.0146394
FYB	ENST00000511927	0.907138796	+	0.00073296	0.01477942
FYB	TCONS_00000200	0.919741649	+	0.00044552	0.01165941
PDE9A	TCONS_00000200	0.943954168	+	0.00012988	0.00684207
IFIT3	ENST00000538202	-0.915456537	-	0.00053219	0.01259604
CDY1B	TCONS_00000200	0.958589452	+	4.57E-05	0.00398362
CHST8	TCONS_00000200	0.935124216	+	0.00021484	0.0083889
GPM6B	TCONS_00000200	0.905530823	+	0.0007771	0.01536674
GLIS1	TCONS_00000200	0.93607683	+	0.00020419	0.00810178
PDE9A	TCONS_00000200	0.924754763	+	0.00035728	0.01064919
GFRA2	TCONS_00000200	0.943504246	+	0.0001335	0.00684207
RPAP3	TCONS_00000200	0.909969615	+	0.00065957	0.01386784
GRTP1	TCONS_00000200	0.970195284	+	1.46E-05	0.00275689
FBXO46	TCONS_00000200	0.946143324	+	0.00011322	0.00618922
ATG16L1	TCONS_00000200	0.976522397	+	6.38E-06	0.00174407
DEFA3	TCONS_00000200	0.982530564	+	2.28E-06	0.00093519
CDC14A	TCONS_00000200	0.920904958	+	0.00042382	0.01133258
MBD6	TCONS_00000200	0.959732289	+	4.15E-05	0.00398362
ZNF185	TCONS_00000200	0.91136266	+	0.00062541	0.01326625
RTL1	TCONS_00000200	0.918489218	+	0.00046974	0.01200679
RAB34	TCONS_00000200	0.937484392	+	0.00018915	0.00794817
CACFD1	TCONS_00000200	0.938497832	+	0.00017881	0.00785493
NR1I3	TCONS_00000200	0.926228825	+	0.00033387	0.01064919

PAX8	TCONS_00000200	0.913494719	+	0.00057555	0.01277452
KRTAP9-4	TCONS_00000200	0.912268428	+	0.00060387	0.01326365
IDH3B	TCONS_00000200	0.966812677	+	2.12E-05	0.00348086
PPA2	TCONS_00000200	0.965987336	+	2.31E-05	0.00355341
KRT6B	TCONS_00000200	0.913838283	+	0.00056779	0.01277452
METTL1	ENST00000511927	0.928596468	+	0.00029855	0.00988264
TBX5	TCONS_00000200	0.916666584	+	0.00050662	0.01236248
ZBTB22	ENST00000511927	0.933095542	+	0.0002388	0.0087268
ZBTB22	TCONS_00000200	0.906020038	+	0.00076348	0.01526957
ZBTB22	ENST00000538202	-0.903417056	-	0.00083789	0.01580464
HOPX	ENST00000511927	0.915441552	+	0.00053252	0.01259604
HSPA8	TCONS_00000200	0.930452088	+	0.00027277	0.00931959
RMND1	TCONS_00000200	0.911356253	+	0.00062556	0.01326625
LAMB2	TCONS_00000200	0.925451355	+	0.00034608	0.01064919
CCDC27	TCONS_00000200	0.98605712	+	1.04E-06	0.00058541
CFHR1	TCONS_00000200	0.916433922	+	0.00051147	0.01236248
ALPP	TCONS_00000200	0.95574652	+	5.75E-05	0.00413941
FAM181B	TCONS_00000200	0.943516738	+	0.0001334	0.00684207
LDLR	TCONS_00000200	0.908474303	+	0.00069766	0.0143021
CLDN4	TCONS_00000200	0.916498022	+	0.00051013	0.01236248
DCN	TCONS_00000200	0.932329165	+	0.00024832	0.0087268
C1orf51	TCONS_00000200	0.905381866	+	0.00078128	0.01536674
TMC4	TCONS_00000200	0.948102304	+	9.96E-05	0.00575086
ZNF79	TCONS_00000200	0.973031206	+	1.03E-05	0.00254124
CBY3	TCONS_00000200	-0.904164033	-	0.00081604	0.01575813
SPCS1	TCONS_00000200	-0.900069757	-	0.00094083	0.01607248
CLCN2	TCONS_00000200	-0.929380114	-	0.00028746	0.00968707
RASGRP4	ENST00000421997	0.952496996	+	7.34E-05	0.00488162
RASGRP4	ENST00000538202	0.941592814	+	0.00014971	0.00711565
RAC2	TCONS_00000200	-0.918302211	-	0.00047344	0.01200679
RPGRIP1L	ENST00000538202	0.900830065	+	0.00091671	0.01604338
SLC4A1	TCONS_00000200	-0.913456862	-	0.00057641	0.01277452
ZNF846	TCONS_00000200	-0.900480034	-	0.00092776	0.01604338
TMEM55B	TCONS_00000200	-0.951021138	-	8.16E-05	0.00528269
FBLIM1	TCONS_00000200	-0.950181997	-	8.65E-05	0.00545802

KLF4	TCONS_00000200	-0.900655191	-	0.00092222	0.01604338
RPL13A	TCONS_00000200	-0.941139423	-	0.00015375	0.00711565
CDK16	TCONS_00000200	-0.914036275	-	0.00056334	0.01277452
MAZ	TCONS_00000200	-0.922263931	-	0.00039942	0.01091744
FAM63A	ENST00000538202	0.911789537	+	0.00061519	0.01326625
UQCRQ	TCONS_00000200	-0.931909404	-	0.00025365	0.00878846
TMEM251	TCONS_00000200	-0.903290597	-	0.00084163	0.01580464
CFD	TCONS_00000200	-0.969581366	-	1.57E-05	0.00275689
MDK	TCONS_00000200	-0.965051787	-	2.54E-05	0.00367419
FLT1	ENST00000538202	0.933528859	+	0.00023353	0.0087268
ARPC2	TCONS_00000200	-0.957679895	-	4.93E-05	0.00398362
GNRH2	TCONS_00000200	-0.954352719	-	6.40E-05	0.00437209
CEP120	ENST00000511927	-0.933462698	-	0.00023433	0.0087268
UQCRC1	TCONS_00000200	-0.903435363	-	0.00083735	0.01580464
GYPC	TCONS_00000200	-0.942765547	-	0.00013961	0.00686889
FPGS	TCONS_00000200	-0.902422423	-	0.00086761	0.01584335
FAM170B	TCONS_00000200	-0.969713197	-	1.55E-05	0.00275689
MCTP1	TCONS_00000200	-0.963258744	-	3.02E-05	0.00374754
SLC44A2	TCONS_00000200	-0.942774243	-	0.00013954	0.00686889
ALPPL2	TCONS_00000200	-0.905176093	-	0.00078708	0.01536674
PILRB	TCONS_00000200	-0.971942058	-	1.19E-05	0.00265074
PFDN1	TCONS_00000200	-0.923581858	-	0.00037671	0.01064919
TCHP	TCONS_00000200	-0.937736422	-	0.00018654	0.00794817
CERS2	ENST00000538202	0.902453993	+	0.00086666	0.01584335
PHYHIP	ENST00000421997	0.940605332	+	0.0001586	0.00711565
PHYHIP	ENST00000538202	0.932460446	+	0.00024667	0.0087268
SRPK2	TCONS_00000200	-0.980909587	-	3.11E-06	0.00098736
SIX1	ENST00000511927	-0.936169674	-	0.00020317	0.00810178
SIX1	ENST00000538202	0.926319271	+	0.00033247	0.01064919
CCBE1	TCONS_00000200	-0.940552147	-	0.00015909	0.00711565
MON1A	TCONS_00000200	-0.925377357	-	0.00034725	0.01064919
ZFPM2	TCONS_00000200	-0.914082584	-	0.00056231	0.01277452
MARS2	TCONS_00000200	-0.937268966	-	0.0001914	0.00794817
TMEM201	TCONS_00000200	-0.924105266	-	0.00036795	0.01064919
DYRK1B	TCONS_00000200	-0.932749029	-	0.00024307	0.0087268

AKR7A2	TCONS_00000200	-0.94120604	-	0.00015315	0.00711565
BYSL	ENST00000511927	-0.900521937	-	0.00092643	0.01604338
BYSL	ENST00000421997	0.9880415	+	6.09E-07	0.00049907
BYSL	ENST00000538202	0.985506611	+	1.19E-06	0.00058541
WNT4	ENST00000538202	0.908898121	+	0.00068671	0.01419594
BMF	TCONS_00000200	-0.957446503	-	5.02E-05	0.00398362
CCNL2	ENST00000511927	-0.92107875	-	0.00042064	0.01133258
CCNL2	ENST00000538202	0.914494606	+	0.00055316	0.01277452

Note: PCC: Pearson correlation coefficient.

Supplemental Table 2: Top five GO entries for enrichment scores related to biological process, cellular component, and molecular function

Ontology	Count	Pop. Hits	List. Total	Pop. Total	Fold. [illegible]	Pvalue	FDR	Enrichment. [illegible]	Gene.Ratio	GENES
Biological process	3	37	135	16672	10.01321321	0.003299033	1	2.48161333	0.022222222	MSTN//USP2//MAPK11
Biological process	6	179	135	16672	4.139540658	0.003378327	1	2.471298318	0.044444444	POP7//GTPBP3//PPA2/
Biological process	2	11	135	16672	22.45387205	0.003412391	1	2.466941149	0.014814815	CD300LG//GPIHBP1
Biological process	3	39	135	16672	9.4997151	0.003834907	1	2.416245151	0.022222222	FGF7//REG3A//FERMT1
Biological process	3	39	135	16672	9.4997151	0.003834907	1	2.416245151	0.022222222	MSTN//USP2//MAPK11
Cellular component	81	7919	144	17942	1.274450688	0.002230867	0.586778053	2.65152635	0.5625	DHRS4//CKMT1B//CMC
Cellular component	33	2567	144	17942	1.601756265	0.003609481	0.586778053	2.442555238	0.229166667	HAVCR2//GPIHBP1
Cellular component	2	12	144	17942	20.7662037	0.004005086	0.586778053	2.397388123	0.013888889	ICAM2//SPN
Cellular component	2	12	144	17942	20.7662037	0.004005086	0.586778053	2.397388123	0.013888889	ICAM2//SPN
Cellular component	3	40	144	17942	9.344791667	0.004025424	0.586778053	2.395188386	0.020833333	BIN1//LDLR//GPIHBP1
Molecular function	2	14	133	16309	17.51772288	0.005633138	1	2.249249591	0.015037594	HBA1//MB
Molecular function	2	20	133	16309	12.26240602	0.011391962	1	1.943401473	0.015037594	CDK13//USP2
Molecular function	3	63	133	16309	5.83924096	0.014750717	1	1.831186869	0.022556391	ATP5G2//ATP7B//ATP1B3
Molecular function	3	63	133	16309	5.83924096	0.014750717	1	1.831186869	0.022556391	FXN//IREB2//MOCS1
Molecular function	3	63	133	16309	5.83924096	0.014750717	1	1.831186869	0.022556391	FXN//IREB2//MOCS1

Note: The *P* value represents the significance of differential gene enrichment in GO entries. The smaller the *P* value is, the more meaningful the GO entry ($P \leq 0.05$). Enrichment score = - log10 (*P*-value).

Supplemental Table 3: Top 10 GO entries for enrichment scores related to biological process

GO.ID	Term	Ontology	Count	Pop. Hits	Pop. Total	Fold.	Pvalue	FDR	Enrichment.	Gene.Ratio	GENES
GO:0045843	Negative regulation of striated muscle tissue	Biological process	3	37	16672	10.01321321	0.003299033	1	2.48161333	0.022222222	MSTN//USP2//MAPK11
GO:0006399	TRNA metabolic	Biological process	6	179	16672	4.139540658	0.003378327	1	2.471298318	0.044444444	POP7//GTPBP3//PPA2//QARS//CARS//GRSF1
GO:0045056	Transcytosis	Biological process	2	11	16672	22.45387205	0.003412391	1	2.466941149	0.014814815	CD300LG//GPIHBP1
GO:0043616	Keratinocyte proliferation	Biological process	3	39	16672	9.4997151	0.003834907	1	2.416245151	0.022222222	FGF7//REG3A//FERMT1
GO:0048635	Negative regulation of muscle organ development	Biological process	3	39	16672	9.4997151	0.003834907	1	2.416245151	0.022222222	MSTN//USP2//MAPK11
GO:0050821	Protein stabilization	Biological process	5	128	16672	4.824074074	0.003861858	1	2.413203737	0.037037037	GPIHBP1//ATP1B3//WFS1//USP2//BAG3
GO:0051546	Keratinocyte migration	Biological process	2	12	16672	20.58271605	0.004073212	1	2.390062949	0.014814815	FGF7//FERMT1
GO:1901862	Negative regulation of muscle tissue development	Biological process	3	40	16672	9.262222222	0.004121543	1	2.384940176	0.022222222	MSTN//USP2//MAPK11
GO:0048642	Negative regulation of skeletal muscle tissue	Biological process	2	13	16672	18.9994302	0.004788353	1	2.319813863	0.014814815	MSTN//USP2

GO:0046916	Cellular transition metal ion homeostasis	Biological process	4	85	16672	5.811590414	0.005018486	1	2.29942724	0.02962963	ATP7B//FXN//IREB2//SLC30A1

Supplemental Table 4: GO entries related to immune inflammatory responses in biological processes

GO code	Entry	Enrichment score	P-value	GENES
GO:0042130	Negative regulation of T cell proliferation	2.187626037	0.006491932	SPN//CD276//HAVCR2
GO:0032722	Positive regulation of chemokine production	2.112920501	0.007710446	EGR1//TRIM32//HAVCR2
GO:0032703	Negative regulation of interleukin-2 production	1.992690951	0.010169721	CD276//HAVCR2
GO:0032945	Negative regulation of mononuclear cell proliferation	1.916020422	0.012133318	SPN//CD276//HAVCR2
GO:0050672	Negative regulation of lymphocyte proliferation	1.916020422	0.012133318	SPN//CD276//HAVCR2
GO:0032642	Regulation of chemokine production	1.750814239	0.017749485	HAVCR2//EGR1//TRIM32
GO:0032602	Chemokine production	1.654187048	0.022172413	HAVCR2//EGR1//TRIM32
GO: 0002707	Negative regulation of lymphocyte-mediated immunity	1.585631128	0.025963837	SPN//HAVCR2
GO: 0002823	Negative regulation of immune response	1.559875771	0.027550167	SPN//HAVCR2
GO:0050868	Negative regulation of T cell activation	1.538804252	0.028919831	SPN//CD276//HAVCR2
GO: 0002820	Negative regulation of adaptive immune response	1.487651551	0.032534823	SPN//HAVCR2
GO:0032732	Positive regulation of interleukin-1 production	1.465095307	0.034269257	EGR1//HAVCR2
GO:1903038	Negative regulation of leukocyte cell-cell adhesion	1.44835095	0.03561632	SPN//CD276//HAVCR2
GO:0042102	Positive regulation of T cell proliferation	1.400436155	0.039770756	SPN//CD276//HAVCR2
GO: 0002704	Negative regulation of leukocyte-mediated immunity	1.343004938	0.045393646	SPN//HAVCR2

Note: The *P* value represents the significance of differential gene enrichment in GO entries. The smaller the *P* value is, the more meaningful the GO entry ($P \leq 0.05$

Supplemental Table 5: Related biological pathways

Pathway ID	Definition	Original Website	Fisher-*P*-value	Selection Counts	Selection Size	Count	Size	FDR	Enrichment Score	Gene Ratio	Genes
Hsa04978	Mineral absorption - Homo sapiens	Http://www.genome.jp/kegg-bin/show_pathway?h	0.001207251	4	65	52	7161	0.3730407	2.918202	0.061538	ATP1B3//CLCN2//SLC26A9//SLC30A1
Hsa00790	Folate biosynthesis - Homo sapiens	Http://www.genome.jp/kegg-bin/show_pathway?hsa00790+250+4337	0.007894111	2	65	15	7161	1	2.102697	0.030769	ALPP//MOCS1
Hsa04933	AGE-RAGE signalling pathway in diabetic complications	Http://www.genome.jp/kegg-bin/show_pathway?h sa04933+1288+1958+5600+4772	0.01308147	4	65	101	7161	1	1.883343	0.061538	COL4A6//EGR1//MAPK11//NFATC1
Hsa01524	Platinum drug resistance - Homo sapiens (human)	Http://www.genome.jp/kegg-bin/show_	0.03032949	3	65	75	7161	1	1.518135	0.046154	ATP7B//GSTM1//MSH6
Hsa04514	Cell adhesion molecules (CAMs) - Homo sapiens	Http://www.genome.jp/kegg-bin/show_pathway?h sa04514+80381+3384+4359+6693	0.04219688	4	65	145	7161	1	1.37472	0.061538	CD276//ICAM2//MPZ//SPN
